# Are abstract action words embodied? An fMRI investigation at the interface between language and motor cognition

**DOI:** 10.3389/fnhum.2013.00125

**Published:** 2013-04-09

**Authors:** Katrin Sakreida, Claudia Scorolli, Mareike M. Menz, Stefan Heim, Anna M. Borghi, Ferdinand Binkofski

**Affiliations:** ^1^Division of Clinical and Cognitive Neurosciences, Department of Neurology, Medical School, RWTH Aachen UniversityAachen, Germany; ^2^Department of Psychology, University of BolognaBologna, Italy; ^3^Department of Systems Neuroscience and Neuroimage Nord, University Medical Center Hamburg EppendorfHamburg, Germany; ^4^Section Structural Functional Brain Mapping, Department of Psychiatry, Psychotherapy and Psychosomatics, Medical School, RWTH Aachen UniversityAachen, Germany; ^5^Institute of Neuroscience and Medicine (INM-1), Research Centre JülichJülich, Germany; ^6^Institute of Cognitive Sciences and Technologies, National Research CouncilRome, Italy; ^7^Institute of Neuroscience and Medicine (INM-4), Research Centre JülichJülich, Germany; ^8^Translational Brain Medicine, Jülich Aachen Research AllianceJülich, Germany

**Keywords:** language comprehension, abstract, concrete, fMRI, sensorimotor cortex

## Abstract

The cognitive and neural representation of abstract words is still an open question for theories of embodied cognition. Generally, it is proposed that abstract words are grounded in the activation of sensorimotor or at least experiential properties, exactly as concrete words. Further behavioral theories propose multiple representations evoked by abstract and concrete words. We conducted a functional magnetic resonance imaging (fMRI) study to investigate the neural correlates of concrete and abstract multi-word expressions in an action context. Participants were required to read simple sentences which combined each concrete noun with an adequate concrete verb and an adequate abstract verb, as well as an adequate abstract noun with either kind of verbs previously used. Thus, our experimental design included a continuum from pure concreteness to mere abstractness. As expected, comprehension of both concrete and abstract language content activated the core areas of the sensorimotor neural network namely the left lateral (precentral gyrus) and medial (supplementary motor area) premotor cortex. While the purely concrete multi-word expressions elicited activations within the left inferior frontal gyrus (pars triangularis) and two foci within the left inferior parietal cortex, the purely abstract multi-word expressions were represented in the anterior part of left middle temporal gyrus that is part of the language processing system. Although the sensorimotor neural network is engaged in both concrete and abstract language contents, the present findings show that concrete multi-word processing relies more on the sensorimotor system, and abstract multi-word processing relies more on the linguistic system.

## Introduction

Embodied and grounded cognition theories such as Theories of Situated Action, Cognitive Linguistics Theories, Cognitive and Social Simulation Theories (for a review, see Barsalou, 2008), are becoming increasingly popular in cognitive neuroscience. This approach extends to different domains (e.g., perception, action, language, decision-making etc.) and crosses different disciplines, from philosophy (e.g., Clark, 1999), developmental psychology (e.g., Smith, 2005), and social psychology (e.g., Semin and Smith, 2008), to computer science and robotics (e.g., Nolfi and Floreano, 2000; Ziemke, 2002). In contrast to the classical cognitivism that is based on representational systems of symbolic information processing, which distinguishes between so-called high and low cognitive processes, embodied views propose that high-level cognitive processes, such as language comprehension, are grounded in the lower-level processes of perception and action. A wide range of publications within the last decade demonstrates this interest in embodied cognition (for an analysis, see Chatterjee, 2010; Gentner, 2010; Jirak et al., 2010).

When embodied cognition approaches are applied to language comprehension, the notion of “simulation” becomes a prominent feature (e.g., Gallese, 2008). Here, simulation refers to the process of internally representing (or simulating) the content that a word or sentence describes. Thus, the simulation process involves the same sensorimotor neural correlates as during the action execution or when interacting with the actual object or entity itself (Zwaan, 2004). Behavioral and neural evidence has reliably shown that the process of language comprehension elicits activations within primary and secondary motor areas, thus prompting an explanation in terms of embodied simulation (for reviews, see Pulvermüller, 2005; Barsalou, 2008; Fischer and Zwaan, 2008; Toni et al., 2008).

Recently, the limitations of embodied motor simulation have been examined by studies using transcranial magnetic stimulation (TMS). Papeo et al. (2011) showed that enhanced TMS-induced motor-evoked potentials do reflect motor simulation, but that these are restricted to the experimental condition when hand-action verbs were presented in first person, i.e., when the self was recruited as agent, compared to third person verbs and non-action verbs. Moreover, a comparable limb-specific effect for processing of hand-action verbs was found when TMS was applied at 500 ms post-stimulus. This finding indicates that the activity of primary motor cortex was involved in post-conceptional processing, which follows the retrieval of motor representations, rather than in initial lexical-semantic processing (Papeo et al., 2009). As such, the extent to which language comprehension is actually embodied is still the focus of intense debate.

In opposition to a strong embodied approach, some authors propose that sensorimotor system activation during language processing is not necessary for comprehension, since this occurs after the context and the meaning of the information has been computed. Hence, those authors suggest a dynamical interaction among the multimodal modules of language, perception, and action (Mahon and Caramazza, 2008; Bedny and Caramazza, 2011) or gradations from embodied to disembodied cognition (Chatterjee, 2010).

Beyond the discussion on embodiment of language comprehension in general, the debate focuses on disentangling concrete vs. abstract word representations. Hence, abstract word semantics constitute a specific challenge for embodied cognition theories (for a recent review, see Pecher et al., 2011). Embodied representations of abstract words are proposed to underlie activation of sensorimotor, or at least experiential properties, exactly as concrete words. In support of this, Glenberg et al. (2008) used combined behavioral and TMS data to demonstrate that abstract transfer sentences (e.g., “to give some news”) activate motor areas in the same way as concrete transfer sentences (e.g., “to give a pizza”; see also Glenberg and Kaschak, 2002). Further evidence was provided by Barsalou and Wiemer-Hastings (2005) who showed that abstract concepts focus rather on settings and events as well as introspective states than on purely perceptual properties. Further, Kousta et al. (2009, 2011) have demonstrated that abstract as compared to concrete words involve more emotional aspects.

An additional proposal is that multiple representations are evoked by words (for a non-embodied version of this view see Dove, 2009). According to the *Language And Situated Simulation* (LASS) theory, the left-hemispheric language areas are mainly involved during superficial linguistic processing. This consists of word recognition and the immediately subsequent generation of associated word forms (Barsalou et al., 2008). These associated words in turn provide a linguistic context that can be sufficient to perform a wide variety of tasks, such as lexical decision-making tasks. Nevertheless, these superficial strategies may prevent deeper conceptual processing. The conceptual content of properties and relations reside in associated simulations (Barsalou et al., 2008) involving bilateral perceptual and motor neural networks. However, these two systems are not modular, rather they interact in a continuous way. Differently from LASS, the *Words As Tools* (WAT) theory suggests that, in simulation, the linguistic form representation is not superficial and does not prevent deeper conceptual processing. According to WAT words can be conceived as tools that are useful in interacting with the world. During language comprehension a combination of both linguistic and non-linguistic sensorimotor experiences is early on activated and weighted depending not just on the task but also on the kind of considered words (Borghi and Cimatti, 2009, 2010). In fact, the WAT proposal differs from the LASS theory as the former ascribes more relevance to different lexical categories within language, e.g., concrete vs. abstract words, whereas the latter focuses more on the different levels of language processing required for the task, e.g., lexical decision vs. conceptual task.

Both LASS and WAT are in line with the *Dual Coding* theory. This approach ascribes the effect according to which concrete words are memorized better than abstract words to the existence of both a linguistic and a sensorimotor imagery code. Both codes would be activated by concrete words, whereas processing of mere verbal information would be necessary for encoding of abstract words (Paivio, 1971, 1986). Recent functional magnetic resonance imaging (fMRI) studies (e.g., Binder et al., 2005; for a review see Sabsevitz et al., 2005) endorse the *Dual Coding* assumption by showing an activation pattern that confines representation of abstract words to the left hemisphere, whereas it is bilateral for processing of concrete words (for contrasting evidence, see Rodríguez-Ferreiro et al., 2011). Moreover, Desai et al. (2010) found pronounced left-hemispheric superior temporal (BA 22) and inferior frontal (BA 44/45/47) areas activated while processing of abstract sentences (e.g., “use the opportunity”), thus, suggesting that abstract words primarily activate and are understood through verbal associations with other words. However, the embodied multiple representations proposals LASS and WAT extend the Dual Coding theory insofar as both linguistic and sensorimotor information are crucial for not just concrete words but also abstract words.

Since concrete and abstract words rely on different acquisition mechanisms (Borghi and Cimatti, 2010; Borghi et al., 2011) linguistic experience with its social aspects is more important for the acquisition of abstract rather than concrete words, given that abstract words refer to more sparse and diverse experiences than concrete ones. Thus, in line with the WAT proposal it can be assumed that the neural language network predominantly supports processing of abstract words, while concrete words are embedded mainly within the sensorimotor neural network. Evidence supporting an assumption of distributed semantic networks was recently provided by several lesion studies (Mårtensson et al., 2011; Arévalo et al., 2012; Kemmerer et al., 2012).

A number of behavioral (e.g., Day, 1979; Chiarello et al., 1987; Deloche et al., 1987), electrophysiological (e.g., Holcomb et al., 1999; Kellenbach et al., 2002; Nittono et al., 2002), and functional imaging (e.g., Kiehl et al., 1999; Perani et al., 1999; Friederici et al., 2000; Grossman et al., 2002; Noppeney and Price, 2004; Binder et al., 2005; Desai et al., 2010) studies have investigated linguistic abstractness, but the majority of these focused only on single word processing. However, it is clear that human communication consists of much more than an individual word, since words are combined in sentences and these in turn lead to the emergence of word meaning.

The aim of our study was to dissociate neural correlates of concrete and abstract multi-word expressions, focusing on natural linguistic stimuli. To this end we experimentally manipulated very simple sentences composed by a concrete vs. abstract noun and verb. To generate a novel experimental design that encompasses a continuum from pure concreteness to mere abstractness, nouns referring to graspable/non-graspable (concrete/abstract) objects or entities were combined with motor/non-motor (concrete/abstract) verbs. Thus, at one end of the spectrum, a combination of a noun referring to a graspable object with a motor verb (CC) generates a concrete meaning. At the other end, a combination of a noun referring to a non-graspable entity with a non-motor verb (AA) leads to an unambiguous abstract content. The mixed combinations (CA, AC) served to further differentiate between the role of verb and noun in abstract contents processing. Our stimuli and the experimental design were the same as those used in previous behavioral (Scorolli et al., 2011) and TMS (Scorolli et al., 2012) studies, the results of which are expanded on in the discussion.

Based on the embodied approach, our first anatomical prediction concerned activations within the sensorimotor neural network during language processing, regardless of mere concrete or abstract content. Against the background of the LASS and the WAT proposals, our second anatomical hypothesis focused on the dissociation of core areas for pure concrete and mere abstract expression: While concrete noun-verb combinations (CC) should activate pronounced sensorimotor areas, both mixed combinations (CA, AC) and abstract noun-verb combinations (AA) should elicit stronger activations within the neural language network, especially semantic processing areas with their crucial role in the representation of concept meaning.

## Materials and methods

### Participants

We obtained written informed consent from 25 participants (16 female, 9 male, age range 20–36 years, mean age 25.2 ± 3.6) prior to the scanning session. All participants were right-handed with a Laterality Index >0.7 (Annett, 1970) and had normal or corrected-to-normal visual acuity. Only native speakers of German participated in the study. The experimental standards were approved by the local ethics committee. Data were handled anonymously.

### Stimulus generation and standardization

**Figure 1:**
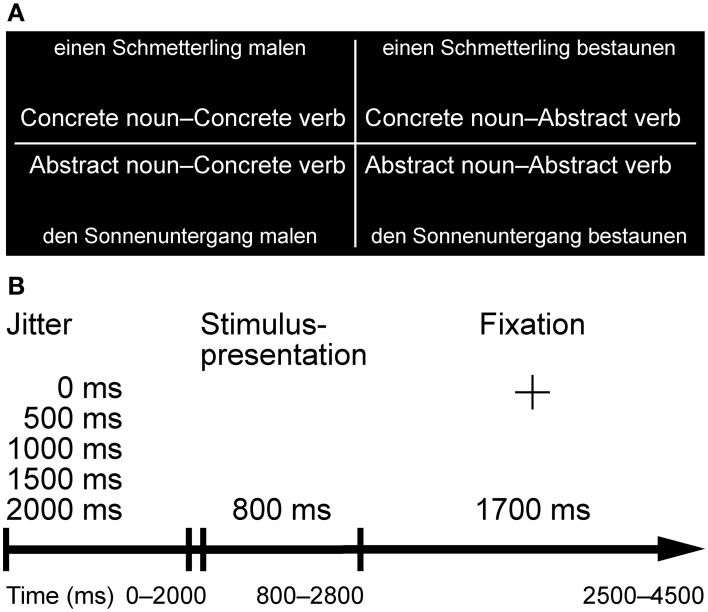
**Experimental design with an exemplary stimuli combination (A) and trial procedure (B).** Each noun referring to a graspable object, preceded by a determinative or non-determinative article, was combined with an adequate motor verb as well as an adequate non-motor verb, and an adequate noun referring to a non-graspable entity was combined with the same verbs previously used, e.g., “einen Schmetterling malen” (to draw a butterfly), CC—“einen Schmetterling bestaunen” (to marvel at a butterfly), CA—“den Sonnenuntergang malen” (to draw the sunset), AC—“den Sonnenuntergang bestaunen” (to marvel at the sunset), AA. Note that according to the German word order the noun is presented first followed by the verb. Due to the variable onset delay (jitter) the trial duration was 2500 ms at minimum to 4500 ms at maximum.

Noun-verb combinations for fMRI stimulus generation included 96 German nouns—48 (concrete) graspable objects and 48 (abstract) non-graspable entities—and 96 German verbs—48 (concrete) motor verbs and 48 (abstract) non-motor verbs—Note that according to the German word order, the noun is presented first followed by the verb. Each noun referring to a graspable object (C), preceded by a determinative or non-determinative article, was combined with an adequate motor verb (C) as well as an adequate non-motor verb (A), and an adequate noun referring to a non-graspable entity (A) was combined with the same verbs previously used, e.g., “einen Schmetterling malen” (to draw a butterfly), CC—“einen Schmetterling bestaunen” (to marvel at a butterfly), CA—“den Sonnenuntergang malen” (to draw the sunset), AC—“den Sonnenuntergang bestaunen” (to marvel at the sunset), AA (see Figure 1A). Thus, 48 quadruples of pairs were created that were formed by two nouns and two verbs each, resulting in 192 noun-verb combination stimuli. This particular paradigm encompasses a concreteness-to-abstractness continuum. Any metaphorical or idiomatic combinations, as for instance “to kick in the dugout” or “to kick the bucket” were avoided.

Twenty German students were asked to judge the familiarity of each noun-verb combination and for the degree of probability they would use it. Ratings were given by making a cross on a continuous line scale (not familiar—very familiar; not probably—very probably). Subsequently, 18 quadruples with lowest scores and highest standard deviations for both familiarity and probability of use were removed, thus, finally 30 quadruples including 120 noun-verb pairs were selected for the fMRI experiment.

Due to the peculiarity of the linguistic material, in a first step, the lexical frequency of all stimuli in both written and spoken German was assessed using the CELEX database (Baayen et al., 1996). The averages for all four stimuli types were above 400/million, i.e., in the range of high-frequency words (concrete nouns: 404/million; concrete verbs: 530/million; abstract nouns: 587/million; abstract verbs: 3132/million). The relatively high frequency of abstract verbs resulted from the item “haben” (to have). Scores were submitted to a 2 (concrete vs. abstract) × 2 (noun vs. verb) ANOVA. Analysis yielded no significant effects [no main effect concrete vs. abstract: *F*_(1, 29)_ = 1.87; *p* = 0.182, no main effect noun vs. verb: *F*_(1, 29)_ = 1.83; *p* = 0.186, no interaction: *F*_(1, 29)_ = 1.52; *p* = 0.228]. In a second step, the frequency of each noun-verb combination in written German was assessed by using the search engine “Google” with each multi-word expression within quotations marks as search terms (updated at March 2012). The 2 (kind of noun: concrete vs. abstract) × 2 (kind of verb: concrete vs. abstract) ANOVA did not show any significant difference across the four conditions [no main effect concrete vs. abstract noun: *F*_(1, 29)_ = 0.09; *p* = 0.763, no main effect concrete vs. abstract verb: *F*_(1, 29)_ = 0.96; *p* = 0.335, no interaction: *F*_(1, 29)_ = 2.01; *p* = 0.167].

Furthermore, in an additional study the linguistic material was standardized for imageability, literality, quantity of motion as well as for age of acquisition. Methods and results are reported in the Supplementary Material to this article and discussed in detail by Scorolli et al. (2011).

### Experimental paradigm and task

Task instructions were implemented in a go-nogo paradigm. Participants were asked to carefully read the 30 visually presented critical quadruples that demanded no motor response. To sustain attention, a button press was required toward oddball multi-word expressions that were 30 combinations of foot-related nouns with foot-related motor verbs, e.g., “einen Ball schießen,” (to kick a ball). Hence, participants were instructed to press a button as fast as possible if the read sentence referred to an action typically performed with the foot and/or leg. Responses were collected with a custom-made four-buttons response-box.

The experimental design included 150 stimuli which were visually presented as white writing on a black background using VisuaStim VGA goggles (Resonance Technology Inc., Northridge, CA, USA) in a pseudo-randomized order. There were five different conditions: noun referring to a graspable object/motor verb (CC), noun referring to a graspable object/non-motor verb (CA), noun referring to a non-graspable entity/motor verb (AC), noun referring to a non-graspable entity/non-motor verb (AA), and oddball condition. Finally, experimental stimuli were supplemented by 15 empty trials used as a low-level baseline (rest condition).

Each trial started with the presentation of a noun and verb simultaneously for a duration of 800 ms, followed by a fixation cross for a duration of 1700 ms. A variable onset delay (jitter) of every stimulus in relation to the acquisition time (0, 500, 1000, 1500, or 2000 ms) produced an oversampling of the actual image acquisition time of 2500 ms by a factor of five, consequently leading to an acquisition sampling rate of 500 ms. Thus, the trial duration was 2500 ms at minimum to 4500 ms at maximum (see Figure 1B).

### Functional localizer task

Subsequent to the main experimental task we ran a finger tapping task in 2 × 4 blocks, which is known to produce robust activation of motor areas (Moritz et al., 2000). Stimuli were green squares presented for 150 ms with either rhythmic or regular intervals (250–1010 ms). Each block started with 23.4 s stimulus presentation, followed by 23.4 s without any visual stimulation ending with a temporary presented yellow square. Participants were instructed to tap a button with their right index finger as accurate as possible synchronous to the duration of the visual pacing (green squares) and to continue tapping throughout the following second unpaced period until the yellow square appeared. Blocks were separated by a 23.4 s rest period.

### Data acquisition

Imaging was performed at 3 T on a Philips magnetic resonance imaging scanner equipped with an 8-channel head coil (Philips). A fast single-shot echo-planar imaging (EPI)-sequence (echo time 30 ms, 90° flip angle, repetition time = 2500 ms) sensitive to blood oxygenation level-dependent (BOLD) contrast was used for acquiring 35 axial slices (240 mm field of view, 80 × 80 pixel matrix, 3 mm thickness, 10% spacing) covering the whole brain. Two functional runs with 330 (localizer task) and 230 (experimental task) T2^*^ scans were performed, with each scan sampling over the 35 slices. The first five volumes of each subject's scan were removed to allow for full T2 saturation. Subsequently, a set of anatomical T1-images (240 mm field of view, 240 × 240 pixel matrix, 164 slices, 1 mm thickness, no gap, echo time = 3.7 ms, repetition time = 8100 ms) was acquired.

### Data analysis

fMRI-data were analyzed using the Statistical Parametric Mapping software SPM8 (Wellcome Department of Cognitive Neurosciences, London, UK) running under Matlab 7.10 (MathWorks Inc., Natick, MA, USA). Spatial preprocessing included realignment to the first scan, coregistration to the T1 anatomical volume images. T1-weighted images were segmented into gray and white matter. This segmentation was the basis for spatial normalization to the Montreal Neurological Institute (MNI) template, which was then resliced and smoothed with a 9 × 9 × 9 mm full width at half maximum Gaussian Kernel filter to improve the signal-to-noise ratio. To correct for low-frequency components, a temporal high-pass filter with a cut-off frequency of 1/128 Hz (=128 s) was applied.

Statistical analyses were performed using the general linear model as implemented in SPM8. In the first-level experimental task analysis for each subject onsets of picture presentation with a duration of 800 ms were used as regressors to the model including the four conditions (CC, CA, AC, and AA) as well as the oddball condition. In the functional localizer task, event related regressors to the model were the response onsets to paced and unpaced—rhythmic and regular—finger tapping.

The second-level analysis was carried out using the flexible factorial design with the first factor SUBJECT and the second factor CONDITION (CC, CA, AC, AA, Tapping). The significance level was set to *p* < 0.05, FWE corrected. Additionally, a cluster size of ≥5 contiguous voxels (40 mm^3^) extended the threshold. The SPM Anatomy toolbox v1.8 (Eickhoff et al., 2005) was employed for anatomical assignments.

## Results

### Behavioral results

Behavioral performance was assessed by correct responses (mean percentage = 95.2%, mean percentage omissions = 4.8%) and reaction times (mean = 966 ms, standard deviation = 407 ms) to oddball multi-word expressions. Thus, as task performance was appropriate, participants' attention was directed toward comprehension of the linguistic material.

Although mean false positive rate was only 2.4% of all nogo trials, most of them occurred when the verb was a concrete one (condition CC: 58.0% of all false positive responses, condition CA: 7.4% of all false positive responses, condition AC: 25.9% of all false positive responses, condition AA: 7.4% of all false positive responses).

### Functional imaging results

#### Whole brain analysis

**Figure 2:**
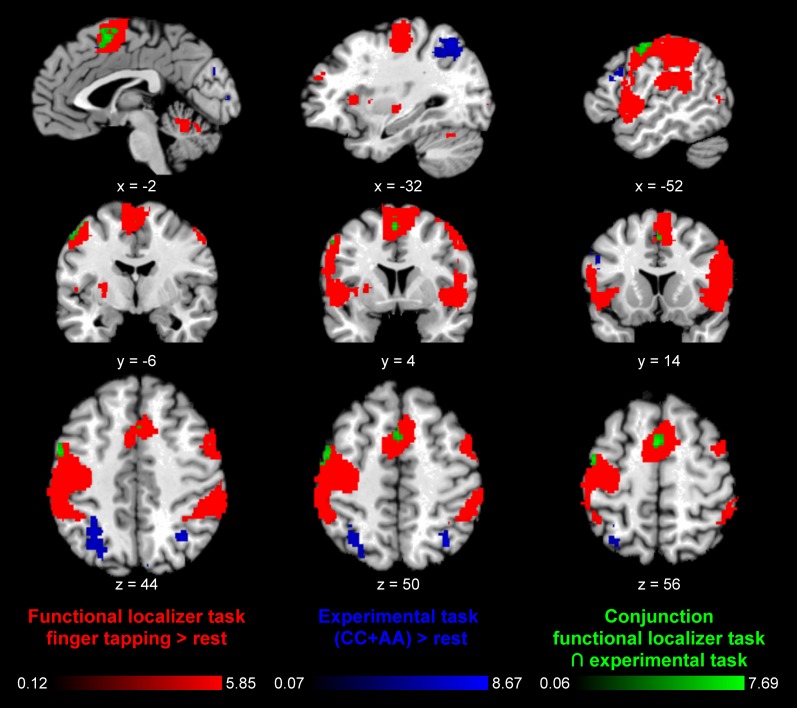
**Absolute activations resulting from functional localizer task and experimental task.** Activations from finger tapping task compared to rest (red), activations from the presentation of abstract and concrete multi-word expressions (CC+AA) in the experimental task compared to rest (blue), and overlapping areas of the functional localizer task and the experimental tasks (green) as revealed by a conjunction analysis. Images were thresholded at *p* < 0.05, FWE corrected for the whole brain volume, superimposed on representative sagittal, coronal and axial slices of the MNI template using the software MRIcron Version 12/2012 (http://www.mccauslandcenter.sc.edu/mricro/mricron/index.html).

The functional localizer task revealed broad activations in left primary and secondary motor cortex including lateral motor/premotor cortex and supplementary motor area, as well as subcortically in the thalamus, the putamen, and the right cerebellum as shown by contrasting the finger tapping periods minus the rest periods. Other activation clusters were located in right postcentral gyrus, right inferior frontal gyrus (pars opercularis), left middle frontal gyrus as well as bilateral in temporal areas and visual cortex (see Figure 2, red-colored, and Table 1).

**Table 1:** **Macroanatomical structure, cytoarchitectonical area (Area_cyto_), percent overlap of cluster with cytoarchitectonical area, cluster size in voxel, MNI coordinates (*x*, *y*, *z*), and maximum *T* value (*T*_max_) of the local maxima for the functional localizer task compared to rest**.

**Local maximum in macroanatomical structure**	**Area_**cyto**_**	**Percent overlap of cluster with cytoarchitectonical area**	**Cluster size (voxel)**	**MNI coordinates**			*****T***_**max**_**
				***x***	***y***	***z***	
L. Precentral gyrus	Area 4a	3.8	5490	−42	−18	54	13.65
R. Inferior frontal gyrus (Pars opercularis)			3719	52	14	0	14.97
L. Supplementary motor area (SMA)	Area 6	44.4	1830	−4	−2	62	14.03
R. Superior temporal gyrus	IPC (PF)	28.4	1472	66	−36	16	11.40
R. Cerebellum	Lobule VI (Hem)	43.6	1157	14	−58	−24	14.12
R. Inferior occipital gyrus			363	42	−84	−6	8.00
L. Putamen			196	−26	−8	6	7.01
L. Middle frontal gyrus			125	−36	50	22	6.76
L. Middle occipital gyrus			101	−24	−90	2	7.04
R. Postcentral gyrus	OP 4	60.5	76	62	−14	20	7.21
L. Thalamus	Th-prefrontal	67.4	38	−12	−18	2	6.46
L. Middle temporal gyrus			12	−52	−68	4	5.70
R. Cerebellum			12	44	−62	−26	6.20
R. Inferior temporal gyrus			8	56	−62	−12	5.93

The significance level was set to p < 0.05, FWE corrected for the whole brain volume. A cluster size of ≥ 5 contiguous voxels (40 mm^*3*^) extended the threshold. Abbreviations: L, left; R, right.

Overlapping areas of the functional localizer task and the experimental conditions as revealed by a conjunction analysis (finger tapping > rest ∩ [CC + AA] > rest) are also depicted in Figure 2, blue color indicating experimental task activations and green color indicating overlapping areas, and listed in Table 2. Two left-lateralized activation clusters encompassed the lateral (precentral gyrus) and medial (supplementary motor area) premotor cortex.

**Table 2:** **Macroanatomical structure, cytoarchitectonical area (Area_cyto_), percent overlap of cluster with cytoarchitectonical area, cluster size in voxel, MNI coordinates (*x*, *y*, *z*), and maximum *T* value (*T*_max_) of the local maxima of the conjunction: functional localizer task (finger tapping > rest) ∩ experimental tasks ([CC + AA] > rest)**.

**Local maximum in macroanatomical structure**	**Area_**cyto**_**	**Percent overlap of cluster with cytoarchitectonical area**	**Cluster size (voxel)**	**MNI coordinates**			*****T***_**max**_**
				***x***	***y***	***z***	
L. Supplementary motor area (SMA)	Area 6	69.1	125	−2	4	56	7.72
L. Precentral gyrus	Area 6	93.3	85	−52	−6	50	7.27

The significance level was set to p < 0.05, FWE corrected for the whole brain volume. A cluster size of ≥ 5 contiguous voxels (40 mm^*3*^) extended the threshold. Abbreviations: L, left; R, right.

**Figure 3:**
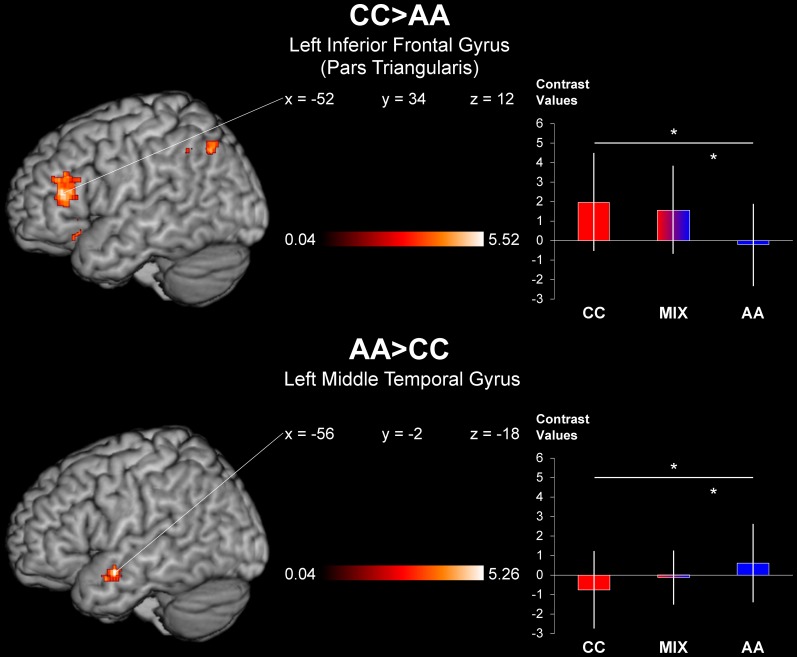
**Direct contrasts of concreteness vs. abstractness.** Differences between processing concrete noun-verb combinations (top panel) compared to abstract noun-verb combinations (bottom panel) and extracted contrast values for the pure abstract, the summarized mixed conditions and the pure abstract condition from defined local maxima. Note that for visualization the statistical images were thresholded at *p* < 0.001, uncorrected, with an extended cluster size of ≥45 contiguous voxels (360 mm^3^), superimposed on the MNI template using the software MRIcron Version 12/2012 (http://www.mccauslandcenter.sc.edu/mricro/mricron/index.html). The contrast values were extracted from the individual beta images and are depicted as group mean with standard deviation of the mean. Asterisks indicate statistical differences of *post-hoc* paired *t* tests (*p* < 0.05, Bonferroni-corrected for multiple comparisons).

As the study focused on differential neural correlates of abstract and concrete contents of language the main effect of interest was achieved by contrasting condition CC (noun referring to a graspable object/motor verb) and condition AA (noun referring to a non-graspable entity/non-motor verb) and vice versa. The direct contrasts CC > AA and AA > CC (*p* < 0.05, FWE corrected for small volumes using the image masks of the SPM Anatomy toolbox v1.8 and a mask of the temporal lobe generated by the WFU PickAtlas Toolbox v3.0.4, respectively) yielded significant activation clusters within a fronto-parietal-temporal network (Figure 3 and Table 3). In the contrast CC > AA the left inferior frontal gyrus (pars triangularis) and two foci within the left inferior parietal cortex were activated, whereas the reverse contrast AA > CC yielded only one suprathreshold activation cluster in the anterior part of left middle temporal gyrus.

**Table 3:** **Macroanatomical structure, cytoarchitectonical area (Area_cyto_), percent overlap of cluster with cytoarchitectonical area, cluster size in voxel, MNI coordinates (*x*, *y*, *z*), and maximum *T* value (*T*_max_) of the local maxima from the direct contrasts of concrete noun-verb combinations compared to abstract noun-verb combinations (CC > AA) and vice versa (AA > CC)**.

**Local maximum in macroanatomical structure**	**Area_**cyto**_**	**Percent overlap of cluster with cytoarchitectonical area**	**Cluster size (voxel)**	**MNI coordinates**			*****T***_**max**_**
				***x***	***y***	***z***	
**CC > AA**							
L. Inferior frontal gyrus (Pars triangularis)	Area 45	63.3	207	−52	34	12	5.55
L. Inferior parietal lobule	hIP1	70.2	80	−36	−50	40	4.89
L. Inferior parietal lobule	IPC (PGa)	54.7	59	−34	−68	42	4.57
**AA > CC**							
L. Middle temporal gyrus^*^			45	−56	−2	−18	5.28

The significance level was set to p < 0.05, FWE corrected for small volumes using the image masks of the SPM Anatomy toolbox v1.8 (Eickhoff et al., 2005). A cluster size of ≥45 contiguous voxels (360 mm^*3*^) extended the threshold. Abbreviations: L, left; R, right.

* *Note that due to the non-availability of a cytoarchitectonical map for that area a mask of the temporal lobe was generated using the WFU PickAtlas Toolbox v3.0.4 (Maldjian et al., 2003) which was applied within the small volume correction and improved the significance*.

#### Regions of interest analysis

The statistical comparisons of the mixed conditions with the pure conditions (CA > CC, AC > CC, CA > AA, AC > AA) showed no significant effects at the chosen threshold. However, to evaluate the effect sizes for the mixed conditions, the group-averaged contrast values of the maximally activated voxel of the frontal (CC > AA) and temporal (AA > CC) activation cluster were statistically compared between all four conditions using repeated-measures ANOVAs and are also displayed in Figure 3. A significant main effect were analyzed within both regions of interest, i.e., left inferior frontal gyrus [*F*_(3, 72)_ = 14.27; *p* < 0.001] and left middle temporal gyrus [*F*_(3, 72)_ = 6.77; *p* < 0.001]. As condition CA did not significantly differ from condition AC in both of the activation peaks, both mixed conditions were combined by averaging. Thus, *post-hoc* paired *T*-tests were calculated for comparisons of three conditions (CC, MIX, AA) with an adjusted significance level to *p* = 0.05 (corresponding to uncorrected *p* = 0.016) by applying a Bonferroni-correction for multiple comparisons (here three). The analysis yielded a significant difference between the MIX condition vs. the AA condition in both regions of interest [left inferior frontal gyrus: *T*_(24)_ = 4.64; *p* < 0.001, left middle temporal gyrus: *T*_(24)_ = 3.16; *p* = 0.004]. The comparison of the MIX condition vs. the CC condition were not significant in the region of interest left inferior frontal gyrus [*T*_(24)_ = 1.71; *p* = 0.101], whereas the differences in contrast values between the MIX and the CC condition in the region of interest left middle temporal gyrus did not reach the corrected significance threshold [*T*_(24)_ = 2.17; *p* = 0.040]. Note that based on the above mentioned main effects, condition CC differed significantly from condition AA in all clusters, i.e., left inferior frontal gyrus [*T*_(24)_ = 5.34; *p* < 0.001] and left middle temporal gyrus [*T*_(24)_ = 3.21; *p* = 0.004].

## Discussion

Embodied cognition theories propose that during language comprehension an internal simulation of the content of the word or sentence occurs. Thus, involvement of the same sensorimotor neural network is assumed during the simulation process as while interacting with an object or entity or while executing the action, the word refers to Zwaan (2004).

One of the core open questions in this area concerns the difference in neural representations of concrete and abstract words, as for instance “cake” vs. “theme.” This functional imaging study addressed this question by presenting participants with combinations of nouns referring to graspable/non-graspable objects/entities and motor/non-motor verbs within a concreteness-to-abstractness continuum in order to generate a novel experimental design which also allows to differentiate between the role of verbs and nouns in abstract contents processing.

Firstly, our imaging results replicate previous findings that demonstrate the involvement of motor areas in language comprehension. In contrast to previous studies we chose regular and rhythmic paced and unpaced finger-tapping as a functional localizer. It is important to note that this task excited both primary motor areas and adjacent regions, which are referred to as secondary motor areas that are consequently involved in action observation and language processing. Results show a significant overlap of activations that were evoked by the functional localizer task and also as a result of perceiving abstract and concrete multi-word expressions. The regions involved included the left lateral (precentral gyrus) and medial (supplementary motor area) premotor cortex.

Importantly, our first anatomical prediction was confirmed for the motor areas recruited by language stimuli. Specifically, the activations elicited by the concrete and abstract multi-word expressions were not significantly different from each other within the motor areas identified by the localizer task. Hence, processing of both concrete and abstract language content is crucially supported by the sensorimotor neural network.

Regarding our second anatomical hypothesis, which focused on the dissociation of core areas for both pure concrete and mere abstract multi-word expressions, considerable differences in neural correlates were identified in the present study. Processing of concrete compared to abstract multi-word content predominantly employed a fronto-parietal network, which is a well-known circuit for object perception and manipulation (see early fMRI studies by Binkofski et al., 1999; Buccino et al., 2001). This shows that this network could also be activated by reading nouns that refer to graspable objects, which might reflect the possible nature of the interaction with the object. Conversely, processing of abstract noun-verb combinations compared to concrete language content showed a pronounced activation in the left anterior middle temporal gyrus. Crucially this area is close to the language processing system (see Price, 2010).

The finding of left-lateralized contribution of middle temporal gyrus to abstract rather than to concrete words comprehension is in line with several functional imaging studies on the ability to mentally imagine concrete vs. abstract nouns (Mellet et al., 1998; Sabsevitz et al., 2005). In previous studies, anterior middle temporal gyrus has consistently been activated during categorization of unique entities, such as famous faces (Sergent et al., 1992; Gorno-Tempini et al., 1998; Leveroni et al., 2000; Martin and Chao, 2001). In recent TMS studies, left posterior middle temporal gyrus was shown to be a crucial part of a distributed network for semantic control (Whitney et al., 2011, 2012). Interestingly, (posterior) middle temporal gyrus was found to be recruited during processing of semantic jokes (Goel and Dolan, 2001), while the same area is also involved in control for action rationality, as for instance when goal-directed actions are violated due to contextual constraints, as in the presence of an obstacle (Jastorff et al., 2011). The responsiveness to the violation of contextual constraints seem to be critical in communication and in social interaction. In turn, this appears to be in line with the WAT proposal that the social context of language acquisition impacts upon representations of abstract rather than concrete language content.

Crucially, the pronounced involvement of left middle temporal gyrus in abstract language processing supports multiple representation theories like LASS and WAT. This is insofar as although concrete and abstract language content engages the sensorimotor neural network, abstract word processing relies more on the linguistic neural system. The idea of parallel systems, the language and the motor one, for preferentially processing concreteness and abstractness has been already discussed by Scorolli et al. (2011). Their reaction time study used the same linguistic material but implemented a sentence evaluation task. Reaction times were significantly faster while processing pure concrete and pure abstract language content (CC, AA) compared to the mixed conditions CA and AC (see also a recent TMS study with the same paradigm, Scorolli et al., 2012). However, disambiguation of processing within linguistic neural system preceding or resulting from sensorimotor processing is constrained by the poor temporal resolution of fMRI data in principle.

As well as mere abstract language content, the mixed combinations (CA, AC) also characterized representations of concept meaning. Thus, according to our second anatomical hypothesis, the neural language network was also assumed to be involved while semantically processing nouns referring to a graspable object combined with non-motor verbs or nouns referring to a non-graspable entity combined with motor verbs. Indeed, the mixed combinations reveal activations of the same areas as recruited by the pure abstract and concrete condition, even if to a greater or lesser extent. To point out in detail, analysis of effect sizes within the frontal and temporal regions of interest showed that the pure concrete condition (CC) did not differ from the summarized mixed conditions (CA + AC), but rather conversely, the pure abstract condition (AA) differed significantly from the summarized mixed conditions (CA + AC). Consequently, if either the noun or the verb becomes an abstract meaning, semantic processing predominantly changes to ambiguity. These results in part support the ability of our paradigm to implement a continuum from concreteness to abstractness, and thus, future research could adopt a similar approach by finding appropriate linguistic material.

One point is worth of notice. Even if the present study did not aim to investigate the different representations elicited by multi-word expressions with various degrees of metaphoricity, we cannot exclude a variation of our materials along the continuum of literal-figurative language. Related theories like the coarse-semantic-coding theory (Jung-Beeman, 2005) and the graded salience hypothesis (Giora, 1997) focused directly on the different neural underpinnings of literal and figurative language. The former suggests a right hemispheric advantage for tasks requiring both the integration of distant semantic concepts and for the understanding of figurative language whereas the left hemisphere, instead, would be specialized in analytic tasks that require the processing of literal semantic associations. The latter assumes the dimension of novelty-conventionality to be more salient than the distinction between literal and metaphorical language. Both theories predict that literal language is processed primarily in the dominant left hemisphere, while novel figurative language has faster access to the right hemisphere. But the two theories differ with respect to conventional figurative expressions: According to the coarse-semantic-coding theory the right hemisphere is rather involved in conventional metaphors than in literal expressions, while the graded salience hypothesis proposes conventional metaphors to be rather processed in the left hemisphere. Due to inconsistent evidence provided by functional imaging studies, Bohrn et al. (2012a) conducted a meta-analysis on neuroimaging studies and have found asides bilateral frontal activations the left middle temporal gyrus to be involved in figurative as compared to literal language processing and in conventional as compared to novel metaphors processing, in line with the graded salience hypothesis. The authors concluded that literal and figurative language processing elicit shared neural correlates, but figurative language requires more cognitive resources to integrate words at the phrase or sentence level, thus, recruit more widespread activations. In sum, the meta-analytic results are convergent with our finding of distinct left lateralized activation within the middle temporal gyrus for abstract sentences, which to some extent resemble conventional metaphors. Interestingly, pronounced extensive left middle temporal gyrus activation was also found by another study by Bohrn et al. (2012b) to be correlated with unfamiliar as compared to familiar proverbs.

Thereby, an interesting idea would be to investigate whether any of the activated areas are triggered just by the noun or the verb depending on the emphasis of concreteness or abstractness on the noun or verb. However, as the noun and the verb were presented simultaneously in our paradigm, this issue remains rather speculative. Interestingly, the study by Rüschemeyer et al. (2007) demonstrated the impact of processing the meaning of the entire word (e.g., “be-greifen,” to comprehend) compared to the meaning of its morphological concrete or abstract components (e.g., “greifen,” to grasp). The components identified by Rüschemeyer et al. (2007) concerned single words rather than word combinations, even though it is possible that the same principle could also be applied to the combination of words. With respect to our results, another interesting open question arises regarding sentence comprehension, i.e., whether a single abstract word, independently of its grammatical class, could shift the whole sentence meaning to a mere abstract one.

Moreover, our imaging data showed a clear pattern of left-lateralized neural correlates associated with both concrete and abstract language content. This is, in part, in line with the findings by Binder et al. (2005), as they reported left-lateralized processing of abstract words and bilateral activations of associative areas during the processing of concrete words. The discrepant evidence might be due to differences in both the tasks and the linguistic materials. Compared to the study of Binder et al. (2005) in which a lexical decision task on word-nonword categorization implies superficial processing of concrete highly imageable words (e.g., “cloud”) vs. abstract low imageable words (e.g., “dogma”) and non-words, our paradigm required semantic processing by combining a noun with a verb within a concreteness-to-abstractness continuum. However, in line with our study Desai et al. (2010) employed a sentence sensibility task implying semantic processing of the linguistic stimuli by manipulation just of the verb meaning (e.g., “use the hammer”—“use the opportunity”), thus, resulting in bilateral activation associated with abstract language, even if stronger for the left hemisphere. Consequently, it remains a matter of debate whether processing of abstract language content rests more on a bilateral than on a left-lateralized neural network.

Similarities in design were highly visible in a study by Christoff et al. (2009) in which anagrams with concrete words (as for instance “desk”), moderately abstract words (as for instance “dance”), and highly abstract words (as for instance “myth”) were used and yielded a functional topography in the prefrontal cortex with relative stronger recruitment of left hemispheric ventrolateral, dorsolateral and rostrolateral prefrontal cortex, respectively, with an curvilinear direction of increase in representational abstraction. Interestingly, even if below the used significance and cluster threshold, we have also found an activation within the rostrolateral prefrontal cortex elicited by the contrast AA > CC (cluster size = 6 voxels; MNI coordinates: *x* = 30, *y* = 46, *z* = −4; maximum *T* value = 3.95). Moreover, our activation was right hemispheric. In contrast to our study, Christoff et al. (2009) employed a task and induced mindsets by cuing the participants to the degree of abstraction of the anagram solution, thus, this might have contributed to the missing significance in our rostrolateral prefrontal activation. Also the application of both a mask of the frontal lobe and a mask of the Brodman area 10, which were generated with the WFU PickAtlas Toolbox v3.0.4 (Maldjian et al., 2003), did not yield significance within a small volume correction. However, the differential representations invoked by reading the word combinations including abstract language content might fit well to the hypothesis of hierarchical processing within the prefrontal cortex (for review, see Badre, 2008; Botvinick, 2008).

## Conclusion

The discussion on cognitive as well as neural representations of concrete vs. abstract linguistic stimuli is still a matter of keen debate. The present fMRI study addressed this question by using a novel paradigm to demonstrate considerable functional dissociations in the neural correlates associated with the concrete and abstract contents of language. In contrast to previous studies that have generally focused on single words in rather superficial lexical-semantic decision-making tasks (for a review, see Sabsevitz et al., 2005), our paradigm implemented semantic processing of multi-word expressions within a concreteness-to-abstractness continuum. To this end, each concrete noun (denoting graspable objects) was combined with an adequate concrete motor verb and an adequate abstract non-motor verb. Likewise, adequate abstract nouns (denoting a non-graspable entity) were combined with either kind of verbs previously used.

First of all, both concrete and abstract multi-word expressions activated the core areas of the sensorimotor neural network. Hence, this is in line with embodied cognition theories. The finding suggests that internal simulation results in the activation of sensorimotor representations, wherein the grounding is in the sensorimotor system for not only concrete but also for abstract language content. In order to show dissociative neural correlates, direct contrasts of pure concrete vs. mere abstract noun-verb combinations and *vice versa* were used. Concrete stimuli revealed adjacent activations to the sensorimotor system whereas abstract stimuli elicited pronounced activation of areas known to underlie lexical and phonological processing. Multiple representations like this in turn are predicted by embodied cognition theories including LASS and WAT proposals. Since both LASS and WAT rely on the idea that multiple representations are activated, both theories are compatible with the results we obtained.

However, only WAT makes specific predictions concerning the difference between concrete and abstract words. As explained in more detail in the introduction, the two theories differ in the role they ascribe to linguistic processes. LASS is focused on lexical vs. conceptual levels of language processing, as it assumes that linguistic processes might be rather superficial, while conceptual processes are not. WAT, instead, does not treat linguistic processing as superficial since it can convey meaning. This theoretical difference has lead the former to put emphasis on the differences between more deep and more superficial tasks and processes (e.g., on the difference between lexical decision and picture naming), without focusing on the differences between semantic categories. In contrast, specific predictions concerning the representation of concrete and abstract words derive from the WAT proposal. More specifically, according to WAT the sensorimotor neural network is engaged by both concrete and abstract words, but in particular by concrete words, while the linguistic neural network is pronounced activated by abstract words. According to WAT, the differences in the representation between these two kinds of words are due to their different acquisition modality, since the absence of a concrete word referent with abstract words needs to be compensated by the use of linguistic labels and explanations. This has been demonstrated through recent behavioral evidence in which new concrete vs. abstract words are learnt (Borghi et al., 2011), but further neural evidence could complement this behavioral data. In sum, even if our findings are compatible with both LASS and WAT theories, the WAT proposal can better predict and account for the dissociation of concrete and abstract language content that we presented in our study.

### Conflict of interest statement

The authors declare that the research was conducted in the absence of any commercial or financial relationships that could be construed as a potential conflict of interest.

## Appendix

### Procedure

Besides selecting the linguistic material with regard to familiarity, probability of use, and lexical frequencies, the noun-verb combinations were standardized concerning imageability, literality, quantity of motion and age of acquisition. Thirty German students of the University of Hamburg were asked to rate on a continuous line scale (scores ranging from 0 to 100) the ease or difficulty with which each multi-word expression evoked mental images (imageability: low imagery rate—high imagery rate), how literally they would take each pair (literality: literal—no literal) as well as whether and to what extent each pair elicited movement information (quantity of motion: not much movement—much movement). Additionally, 10 German students were asked to rate at which age they approximately had learned to use each noun-verb combination (age of acquisition). For each rating, scores' averages (M), and scores' standard deviations (SD) were calculated for each condition: noun referring to a graspable object/motor verb (CC), noun referring to a graspable object/non-motor verb (CA), noun referring to a non-graspable entity/motor verb (AC), noun referring to a non-graspable entity/non-motor verb (AA).

### Results: imageability

The Concrete noun—Concrete verb combinations were judged as the easiest to imagine (*M* = 69.10, *SD* = 12.76), followed by the Concrete noun—Abstract verb combinations (*M* = 52.72, *SD* = 15.80), the Abstract noun—Concrete verb combinations (*M* = 48.53, *SD* = 12.92), and finally by the Abstract noun—Abstract verb combinations (*M* = 45.56, *SD* = 14.51). Therefore, results found the noun to be stronger than the verb in determining the imageability of the whole multi-word expression (see Paivio, 1965).

### Results: literality

The Concrete noun—Abstract Verb combinations were rated as to take most literally (*M* = 18.89, *SD* = 13.72), followed by the Concrete noun—Concrete verb combinations (*M* = 20.22, *SD* = 18.12), the Abstract noun—Abstract verb combinations (*M* = 31.23, *SD* = 19.59), and finally by the Abstract noun—Concrete verb combinations (*M* = 56.95, *SD* = 19.01). Thus, participants judged the multi-word expression of condition CA as most literal and the multi-word expression of condition AC as most metaphorical. Note that the meaning of the concrete verb may be different across condition CC and condition AC depending on the context, e.g., the meaning of the verb “schmieden” (to forge) within the expression “einen Ring schmieden” (to forge a ring) differs from its meaning within the expression “einen Plan schmieden” (to forge a plan). In contrast, the meaning of the concrete noun remains the same across condition CC and condition CA, e.g., “einen Ring schmieden” (to forge a ring)—“einen Ring auswählen” (to select a ring).

### Results: quantity of motion

The Concrete noun—Concrete verb combinations were rated as eliciting most movement information (*M* = 34.29, *SD* = 13.95), followed by the Abstract noun—Concrete verb combinations (*M* = 27.22, *SD* = 12.82), the Abstract noun—Abstract verb combinations (*M* = 17.98, *SD* = 13.87), and finally by the Concrete Noun—Abstract verb combinations (*M* = 13.99, *SD* = 7.39). Thus, multi-word expression of condition CA elicits less movement.

### Results: age of acquisition

The age rated by adults has been demonstrated to be the major independent predictor of the objective age of acquisition indices (Gilhooly and Gilhooly, 1980; Zevin and Seidenberg, 2002). Participants rated the Concrete noun—Concrete verb combinations as the ones they learnt first (*M* = 7.82 years old, *SD* = 2.21), followed by the Concrete noun—Abstract verb combinations (*M* = 8.64 years old, *SD* = 2.55), and finally by both Abstract noun—Concrete verb combinations (*M* = 10.74 years old, *SD* = 1.95) and Abstract noun—Abstract verb combinations (*M* = 10.24 years old, *SD* = 2.35). Hence, based on concrete nouns were learned earlier than abstract nouns results suggest the noun to be critical in language acquisition.
